# Round the Hospitals

**Published:** 1916-12-30

**Authors:** 


### ROUND THE HOSPITALS.

Christmas Day 1916 will be memorable throughout the hospital world as one of the quietest and yet one of the most inspiriting and satisfactory of Christmas Days ever spent in the majority of British hospitals. The cheeriness of the soldier patients, the apprehension of the strenuous times in which we are now living, the note of personal service which rang true throughout our hospital system from end to end, produced an atmosphere of aspiration that will dwell in the memory of British people for the rest of their lives. We rejoice especially that, as recorded elsewhere, the King and Queen, with their children, for the first time, spent some portion of Christmas Day in a great hospital. Queen Alexandra, too, visited the patients in her own hospital in Queen Square and received the Sacrament there, a memorable fact and example which will stir the hospital world to its centre. All these signs are of the first importance, for they emphasise the spirit to which practically the whole nation and Empire are attuned. It has always been a great privilege to spend one's time and thought and energies in work for the sick, especially in a hospital. In this the third year of the war hospital workers have, we believe, realised, more, probably, then ever before, the nobility of their work, the inspiration of their surroundings, and the uplifting nature of the personal service it is their privilege to render continually. Once a hospital worker with the true spirit, and the blessedness it brings to the spiritually-minded will never be lost. It has been a blessed Christmas this year for Editor, readers and workers. We hope 1917 will prove ful of blessings too for all.

---

Throughout the British nursing world it may be welcome news that The Nursing Mirror's invitation to trained nurses to fill in their application form for registration with the College and to send it to the Editor has been welcomed by nurses from one end of the country to the other. Amongst several hundred applications there are representatives from all parts of the country, from John o' Groats to Land's End, from Shetland and many parts of Scotland, as well as England and Ireland. Amongst the applicants for registration with the College are representatives of certificated nurses from the London and St. Bartholomew's Hospitals, the Society for the State Registration of Nurses, English and Scottish Poor-Law nurses, nurses from the great cities of the United Kingdom, and certificate-holders of the Medico-Psychological Association. Some applications have been received from the Front, and others are from nurses who have been trained in India. Here, then, is irrefutable evidence that nursing opinion throughout Great Britain and in various parts of Ireland is genuinely interested in the College of Nursing, its Register, and the Hon. Arthur Stanley's leadership and proposals for the recognition and protection of the nursing profession by statute. It is most satisfactory, though not surprising, that British nurses throughout the United Kingdom should thus unite in defence of their rights and in support of the principles which will give to nurses not only registration but a definite status and statutory recognition for their profession. That profession, too, will henceforth be placed upon a democratic basis, which will secure to the nurses the right to manage their own affairs and to protect their own interests.

---

Now that the actual views and wishes of the general body of nurses throughout the country have been voiced it is essential that every one interested in the College should get to business. Success demands that individually and in combination all must set themselves to organise the nurses and speed them up with their applications for registration with the College. We propose next week to outline a scheme whereby matrons and sisters, who are interested, can render a measure of personal service of the first importance, which will tend to build up the work and strengthen the hands of Mr. Arthur Stanley, the nurses' Parliamentary representative. It is one thing to be a member of the Council of the College and quite another to devote one's whole energies and available time to the work of recruiting so that the College Register may contain at least 20,000 names by the end of February, as it certainly can do, if everybody works with a will. We should welcome an intimation from any matron or sister who would be willing to meet nurses, talk over their difficulties with them, help them in filling up their forms, and generally.

---

Miss Ruth E. Darbyshire, R.R.C., matron since 1913 of St. Mary's Hospital, and principal matron of the 2nd London General Hospital (T.), has been appointed superintendent of Lady Minto's Indian Nursing Association. Miss Darbyshire, who trained at St. Thomas's Hospital, served there first as sister in the operating theatre, and subsequently as sister in charge of the isolation block containing sixty beds. In 1907 she was selected to fill the post of matron to the Derbyshire Royal Infirmary, where she remained till her appointment to the matronship of St. Mary's Hospital, vacated by Miss M. E. Davies' appointment as acting matron of King George Hospital. Miss Darbyshire was awarded the Royal Red Cross, First Class, in June 1916. Miss Darbyshire has proved herself worthy of her training-school in each post she has filled, and the Selection Committee of Lady Minto's Indian Nursing Association are to be congratulated on their choice. The new superintendent should find an extended sphere of usefulness in India, while the change of climate, surroundings, and people cannot fail to prove instructive and full of interest. We wish Miss Darbyshire complete success in the wide field of opportunity which awaits her in the Indian Empire of His Majesty.

