# Erratum

**Published:** 1917-01-06

**Authors:** 


### ERRATUM.

WE regret that, through an oversight, on pages 255 and 272 of last week's issue the Alexandra Hospital for Children is mentioned in error. It should be The Queen Alexandra Military Hospital, Millbank.

